# Does taking statins affect the pathological burden in autopsy-confirmed Alzheimer’s dementia?

**DOI:** 10.1186/s13195-018-0430-7

**Published:** 2018-10-02

**Authors:** Jana Crum, Jeffrey Wilson, Marwan Sabbagh

**Affiliations:** 1 Arizona State University, Tempe, AZ USA; 2 Department of Neurology, Barrow Neurological Institute, Phoenix, AZ USA; 3 Cleveland Clinic Lou Ruvo Center for Brain Health, 888 W. Bonneville Ave, Las Vegas, NV 89106 USA

**Keywords:** Statins, Alzheimer’s disease, Dementia, Post mortem, Senile plaques, Neurofibrillary tangles, Braak stages

## Abstract

**Background:**

The efficacy of cholesterol lowering agents, specifically statins, in slowing the rate of decline of cognitive function in Alzheimer’s disease (AD) patients is not yet fully understood. Our team’s previously published paper showed that patients who used statins demonstrated no increase in cognitive decline in mild cognitive impairment when compared with nonusers. Further, AD patients on statins demonstrated a slight decreasing trend in cognitive decline. The purpose of this study is therefore to investigate the association between stain use in AD confirmed by clinical diagnosis and autopsy and the pathological burden (plaques, tangles, Braak stage). The hypothesis leading this investigation is that prolonged statin use associates with lower AD pathology at autopsy.

**Methods:**

We queried the National Alzheimer’s Coordinating Center (NACC) database for autopsy-confirmed AD cases. Of the Uniform Data Set (UDS) participants who are deceased, 16,163 were diagnosed with dementia at their last UDS visit prior to death, and autopsy data are available for 3945 patients. These patients were then stratified into two groups based upon statin use. The two groups were then analyzed for their pathological AD burden, including total plaques, total tangles, age at death, age of onset, and Braak stage.

**Results:**

NACC data were available for 1816 subjects with clinically and pathologically confirmed AD; 1558 were not on statins and 258 were on statins. No significant differences in age at death, age at onset, Braak stages, mean total tau, and mean total amyloid were found between the two subject groups. When statin use was analyzed by apolipoprotein E (ApoE) genotype carrier statins, the presence of ApoE4 did not influence the effects (or lack thereof) of statin use.

**Conclusions:**

Prolonged statin use in pathologically confirmed AD dementia does not appear to influence the amount of burden of plaques and tangles or Braak stage. These observations were not altered by the presence of absence of ApoE4.

## Background

Alzheimer’s disease (AD) is the sixth leading cause of death in the United States. In 2017, Alzheimer’s dementia or related dementias were the primary cause of death for 33% of seniors who expired [1]. It is projected that by 2050, 14 million Americans will have dementia due to Alzheimer’s disease [1]. Due to the increasing burden Alzheimer’s disease adds to the American healthcare system, more than $2.9 billion of the NIH’s research budget has been allocated to AD and AD-related research in 2017 [2].

Numerous studies have investigated the role that cholesterol and lipoproteins play in AD. The direct effects of plasma cholesterol and related lipoproteins on the incidence and severity of dementia and cognitive decline remain a controversial topic deserving of deeper exploration. Recent discoveries have opened up new lines of inquiry to help the neuroscience community more accurately understand and depict the connection between these two variables. Increasing evidence links brain cholesterol with both plaques and tangles [3]. A positive correlation has been shown between HDL levels and MMSE performance and a negative correlation between LDL levels and immediate and delayed recall [4]. Several epidemiological studies also showed that elevated total serum cholesterol was a significant risk factor for Alzheimer’s disease, regardless of ApoE status [5]. Lowering cholesterol levels via statins is associated with reducing Aβ [6]. Subjects with incident dementia have demonstrated higher total cholesterol at their first visit [7]. Cholesterol levels and atherosclerosis have also been found to associate with Alzheimer’s disease [8]. Because lipoproteins appear to adversely affect cognitive functioning, statins have long been purported to play a role in cognitive decline; however, some within the neuroscience community disagree with the notion that the role between statins and dementia is worthy of further exploration. Our study set out to provide compelling evidence that, rather, the relationship between the two is one which we can no longer afford to overlook in our battle against AD and other related forms of dementia.

Evidence that statins decrease the risk of incident dementia is convincing from an epidemiological standpoint. Some studies show that statin users have a fivefold lower risk of incident Alzheimer’s disease and a threefold lower risk of MCI [7]. Three major clinical trials have investigated the role of statins in cognitive decline. The CLASP study in 2011 assessed the use of simvastatin in probable Alzheimer’s disease. No significant difference in cognitive decline was found between simvastatin and a placebo when measured by the ADAS-Cog [9]. The LEADe trial in 2010 studied atorvastatin therapy in mild to moderate Alzheimer’s disease. Again, no net benefit was found resulting from statin therapy compared with placebo over 72 weeks [10] as measured by the ADAS-Cog and ADAS-CGIC. These two clinical trials contradict the initial findings in 2005 by Sparks et al. [11] that displayed a significantly decreased rate of cognitive decline by atorvastatin on the ADAS-cog and MMSE over 6 months. These values approached those which would have suggested compelling significance at the 12 month mark as well [11]. All three of these trials focused on subjects with Alzheimer’s dementia.

Our team’s previously published study explained that patients who used statins showed no negative effects of statins on cognitive decline in amnestic MCI. Further, MCI subjects on statins demonstrated a slight decrease in cognitive decline [12]. This study builds on these results to pose the question asking whether or not statin use decreases the amount of AD pathology at the time of autopsy.

The connection between statins and AD and other forms of dementia warrants exploration. Many studies have investigated the efficacy of statins in reducing AD diagnosis and progression. The purpose of the study is to determine whether statin use in clinically and autopsy confirmed AD is associated with a lower pathological burden (plaques, tangles, Braak stage). This study was carried out to confirm the hypothesis that chronic statin use associates with less AD pathology at autopsy.

## Methods

### Study sample

All data were obtained from the Uniform Data Set (UDS), the Neuropathology Data Set, and the Researcher’s Data Dictionary—Genetic Data from the National Alzheimer’s Coordinating Center (NACC), a database funded by the National Institute on Aging (NIA); compilation of the data began in September 2015. The dataset includes subjects with a range of cognitive characteristics spanning normal cognition, mild cognitive impairment, and dementia. The demographic, clinical, and specimen data from 39 past and present Alzheimer’s Disease Centers were also analyzed, which enroll and follow subjects with their own various protocols. The UDS was collected via a standardized evaluation of subjects during either an office visit, a home visit, or over telephone conversations with a trained clinician or clinic personnel. The information needed was provided by either the subjects themselves or their informants during annual assessment. Written informed consent was obtained from all subjects and informants. Research using the NACC database was approved by the Institutional Review Board at the University of Washington.

This investigation analyzed data gathered by the NACC, established in 1999 in response to a call for a permanent Alzheimer’s disease data-coordinating center and database. Through making this information available to researchers, the NACC aims to maintain and increase the research capability of the NACC database, facilitate and conduct research using NACC data, collaborate with national or international efforts on AD and other dementias, and maintain the NIA-required administrative coordination of ADC meetings and ADC communications [13].

The NACC’s data request website provided all of the data necessary for this study. The data were compiled by the NACC in a dataset. The proposal posed in the query questioned the effect of statins on the neuropathology of dementia due to Alzheimer’s disease. To facilitate acquisition of pertinent variables, the following keywords were used: “Alzheimer’s disease/LOAD”, “Neuropathology”, “Braak stage”, “APOE”, “Cross-sectional”, and “Statin”. The start date of the UDS (September 2005) was used, and the data freeze includes data through December 2016.

AD participants who came to autopsy were assessed based upon their use of statins (+ or –). Participants were placed in either the statin positive or statin negative group. All statin types were grouped together, and no other medications designed to lower lipids were considered. First, the demographical configuration of the subject was determined by categorizing each subject into groups, such as age at onset, age at death, duration of AD, gender, and education level. Subjects were further stratified by the volume of statin users vs nonusers for each demographical factor. Each subject’s Braak stage, overall amyloid load, and overall tau load were analyzed. These subgroups were then cross-analyzed based upon the internal number of statin users vs nonstatin users.

Linear models were fitted to investigate the effects of group difference on NACC age, duration of AD, age at onset, Braak stage, total amyloid, and tau overall. The predictor in these models was statin use or nonuse, and significance was declared based on a probability of type I error level as 0.05 (*α* = 0.05). The assumptions of normality and independence of outcomes among patients were satisfied. Homogeneity was checked, and no evidence concluded otherwise.

## Results

### Overall demographics

The demographic composition of the subjects analyzed is presented in Table 1. For both statin user and nonuser populations, the median age at time of autopsy was 83 years. In addition, males comprised most of both subject groups, representing 60.47% and 53.98% of statin users and nonusers respectively. A total of 532 (29.5%) subjects obtained a graduate or professional degree (mean 19.7 years of education). Of the 1801 subjects with educational background information available, only 256 (14.8%) took statins as recorded in the NACC database. At each level of educational achievement, nonstatin users vastly outnumbered statin users. Duration of AD and gender illustrated a statistically significant difference, with *p* values of 0.015 and 0.030 respectively, with regards to the amount of statin users vs nonusers.

**Table 1:** Demographics of gender and education by statin

	Nonstatin	Statin
Gender		
Male	841 (54%)	156 (54%)
Female	717 (46%)	102 (39.5%)
Education		
Primary school	129 (8.3%)	19 (7.4%)
High school diploma	310 (20.1%)	54 (21.1%)
Some college	263 (17.0%)	42 (16.4%)
Bachelor degree	382 (24.7%)	70 (27.3%)
Graduate/professional school	461 (29.8%)	71 (27.7%)

### Pathological impact

Pathological load differences between statin users and nonstatin users are presented in Table 2. Braak stage showed no statistically significant difference between statin groups, *p* = 0.292. Neither overall tau nor amyloid load demonstrated a statistically significant difference in subject clusters, p = 0.683 and 0.635 respectively.

**Table 2:** Parameter estimates and significance values for responses by statin regardless of Apo E status

	*N*	Mean	Standard error of the mean	*p* value
Age at death				
Non statin	1558	81.72	0.274	0.324
Statin	258	81.07	0.530	
Duration of AD				
Nonstatin	1520	8.8053	0.11082	0.015
Statin	256	8.0938	0.27669	
Age at onset of AD				
Nonstatin	1520	72.0526	0.29309	0.846
Statin	256	72.1992	v.57622	
Braak stage				
Nonstatin	1552	4.8853	0.03685	0.292
Statin	258	4.7829	0.08682	
Total amyloid load				
Nonstatin	1557	2.3378	0.02420	0.635
Statin	258	2.3682	0.05838	
Total tau load				
Nonstatin	1155	1.9957	0.01036	0.683
Statin	193	1.9845	0.02595	

*AD* Alzheimer’s disease

Table 3 shows how the effect of statins on the parameters explored are influenced by ApoE genotype. In nonstatin users, there were significant differences. Age at death and age of onset were significantly younger for ApoE4 carriers. ApoE4 carriers also had significantly higher CERAD neuritic plaque scores, tau scores, and Braak stages.

**Table 3:** Parameter estimates and significance values for responses by statin inclusive of ApoE status

Statin use		APOE4 Mean		*p* value	Mean difference	Standard error of the difference	95% confidence interval of the difference		
							Lower	Upper	
0									
	Age at death	1.00	79.64						
		0.00	81.86	0.014	−2.228	0.895	−3.999	−0.457	Significant
	Education level	1.00	15.59	0.879	−0.132	0.868	−1.835	1.570	Not significant
		0.00	15.72						
	Estimated onset age	1.00	69.13						
		0.00	72.25	0.002	−3.118	0.992	−5.083	−1.153	Significant
	Tauopathy	1.00	2.01						
		0.00	0.34	0.000	1.672	0.076	1.523	1.820	Significant
	Density of neocortical neuritic plaques (amyloid CERAD score)	1.00	2.60						
		0.00	2.32	0.000	0.271	0.075	0.123	0.419	Significant
	Braak stage	1.00	5.29						
		0.00	4.87	0.000	0.420	0.112	0.199	0.642	Significant
1									
	Age at death	1.00	76.81	0.036	−4.543	2.159	−8.795	−0.291	Significant
		0.00	81.36						
	Education level	1.00	14.13	0.444	−1.598	2.085	−5.704	2.508	Not significant
		0.00	15.72						
	Estimated onset age	1.00	67.19	0.001	−5.346	1.335	–8.104	−2.587	Significant
		0.00	72.53						
	Tauopathy	1.00	2.06						
		0.00	0.37	0.000	1.691	0.183	1.330	2.051	Significant
	Density of neocortical neuritic plaques (amyloid CERAD score)	1.00	2.56	0.393	0.207	0.242	−0.270	0.684	Not significant
		0.00	2.36						
	Braak stage	1.00	5.25	0.167	0.498	0.359	−0.210	1.206	Not significant
		0.00	4.75						

*APO* apolipoprotein, *CERAD* Consortium to Establish a Registry for Alzheimer's Disease

0 means ApoE 4 NON carrier

1 means ApoE 4 Carrier

In statin users, there were also significant differences. Age at death and age of onset were significantly younger in ApoE4 carriers. Tau scores were significantly higher in ApoE4 carriers, but neuritic plaque scores and Braak stages did not differ in the statin group between ApoE4 and non-ApoE4 carriers (Table 4).

**Table 4:** Group statistics

Statin use		APOE4	*N*	Mean	Standard deviation	Standard error of the mean
0						
	Age at death	1.00	99	78.96	8.415	0.846
		0.00	1459	81.16	10.957	0.287
	Education level	1.00	99	15.59	2.966	0.298
		0.00	1459	15.72	8.601	0.225
	Estimated onset age	1.00	99	111.61	185.605	18.654
		0.00	1459	91.61	128.096	3.354
	Tauopathy	1.00	99	2.01	0.267	0.027
		0.00	1459	.34	2.698	0.071
	Density of neocortical neuritic plaques (amyloid CERAD score)	1.00	99	2.60	0.699	0.070
		0.00	1459	2.32	0.983	0.026
	Braak stage	1.00	99	5.29	1.042	0.105
		0.00	1459	4.87	1.488	0.039
1						
	Age at death	1.00	16	76.19	6.036	1.509
		0.00	242	80.60	8.591	0.552
	Education level	1.00	16	14.13	3.074	0.769
		0.00	242	15.72	8.290	0.533
	Estimated onset age	1.00	16	67.19	4.764	1.191
		0.00	242	80.19	84.564	5.436
	Tauopathy	1.00	16	2.06	0.250	0.063
		0.00	242	0.37	2.673	0.172
	Density of neocortical neuritic plaques (amyloid CERAD score)	1.00	16	2.56	0.727	0.182
		0.00	242	2.36	0.950	0.061
	Braak stage	1.00	16	5.25	0.856	0.214
		0.00	242	4.75	1.419	0.091

*APO* apolipoprotein, *CERAD* Consortium to Establish a Registry for Alzheimer's Disease

0 means ApoE 4 NON carrier

1 means ApoE 4 Carrier

## Discussion

This study was carried out to confirm the hypothesis that chronic statin use associates with less AD pathology at autopsy. Based upon the analysis of the NACC dataset and assessment of the AD pathological load by subject statin use, several key findings were identified. Statin users demonstrated no statistically significant differences in any pathological load than nonstatin users. No differences for Braak stages, overall tau, and overall amyloid load were found. Upon further analysis, in each level of overall amyloid level, nonstatin users drastically outnumbered statin users suggesting that statin use in AD is relatively small. Further, we find that the effect of statin use is not confounded or impacted by ApoE carrier status. These data suggest that statin use did not protect against AD pathology in autopsy-confirmed AD dementia cases. Additionally, these data suggest that statins do not negatively impact AD.

An additional finding is that ApoE4 carriers had increases in Braak stages, tau pathology, and amyloid CERAD plaque counts. This is in line with previously published reports [14, 15].

Limitations on generalization of this study are many. First, because it is a case–control study, we rely on what is captured in the database. It is possible that many participants were on statins in the past but are no longer. Second, with our stratification, we could not include the amount of and time for which subjects used statins and the type of statin used. The length of time the statin therapy is employed may contribute to the macroscale impact statins seem to have on the duration of subjects’ AD battle. Also, no comorbidities were taken into consideration. For example, hypercholesterolemia is a risk factor for AD and would alter selection of statin use in that population. The subjects in this study may have had no use for statin intake, therefore the low statin use levels. Another limitation is that neuropathological measures of plaques, tangles, and Braak staging are somewhat limited, the plaque measures in particular as they only tell part of the amyloid story—they represent the insoluble amyloid but not the soluble species, wherein the highly aggregative and arguably more toxic oligomeric forms exist. As such, it is possible that statin exposure is protective against levels of soluble amyloid, wherein amyloid trafficking supported by cholesterol/lipoproteins and some of their receptors (e.g., LRP1 and others thought to be important receptors in active amyloid clearance from the brain) are thought to be important, but these are not variables assessed. Yet another limitation is that statins might exert protective benefits beyond plaques and tangles. Hypoxia and inflammation are two mechanisms that can adversely contribute to cognitive decline and which statins have been claimed to be protective, suggesting that these are also factors that could have explained their associations with clinical data but cannot be excluded from this study.

Future studies should investigate whether the amount of time of statin use impacts on the AD pathological load of subjects.

## Conclusions

Prolonged statin use in pathologically confirmed AD dementia does not appear to influence the amount of burden of plaques and tangles or Braak stage and the effect is not altered by the presence or absence of ApoE4. Our hypothesis that chronic statin use might be associated with lower AD pathological burden was not confirmed despite the large body of evidence that statin use might protect against AD changes. The difference here is that our study was focused on autopsy-confirmed AD and not longitudinal cohorts. This postmortem study indicates that chronic statins do not alter AD pathology at the end of life. Statins might exert beneficial effects earlier in the disease but appear not to impact the pathology when investigated post mortem.

## References

[1] Alzheimer’s Association 2018 Alzheimer’s and Disease Facts and Figures. www.alz.org/media/HomeOffice/facts%20and%20figures/facts-and-figures.pdf.

[2] National Institutes of Health Estimates of Funding for Various Research, Condition, and Disease Categories (RCDC). https://report.nih.gov/rcdc.

[3] Kandiah N, Feldman HH (2009). Therapeutic potential of statins in Alzheimer’s disease. J Neurol Sci.

[4] Sparks DL, Kryscio RJ, Connor DJ (2010). Cholesterol and cognitive performance in normal controls and the influence of elective statin use after conversion to mild cognitive impairment: results in a clinical trial cohort. Neurodegener Dis.

[5] McGuinness B, Craig D, Bullock R, Malouf R, Passmore P (2014). Statins for the treatment of dementia. Cochrane Database Syst Rev.

[6] Chuang C, Lin C, Lin M, Sung F, Kao C (2015). Decreased prevalence of dementia associated with statins: a national population-based study. Eur J Neurol.

[7] Beydoun MA, Beason-Held LL, Kitner-Triolo MH (2011). Statins and serum cholesterol's associations with incident dementia and mild cognitive impairment. J Epidemiol Community Health.

[8] Malfitano AM, Marasco G, Proto MC, Laezza C, Gazzerro P, Bifulco M (2014). Statins in neurological disorders: an overview and update. Pharmacol Res.

[9] Sano M, Bell K, Galasko D (2011). A randomized, double-blind, placebo-controlled trial of simvastatin to treat Alzheimer disease. Neurology.

[10] Feldman HH, Doody RS, Kivipelto M (2010). Randomized controlled trial of atorvastatin in mild to moderate Alzheimer disease: LEADe. Neurology.

[11] Sparks DL, Sabbagh MN, Connor DJ (2005). Atorvastatin therapy lowers circulating cholesterol but not free radical activity in advance of identifiable clinical benefit in the treatment of mild-to-moderate AD. Curr Alzheimer Res.

[12] Smith KB, Kang P, Sabbagh MN, Initiative A’s DN (2017). The effect of statins on rate of cognitive decline in mild cognitive impairment. Alzheimers Dement.

[13] Beekly DL, Ramos EM, Lee WW, Deitrich WD, Jacka ME, Wu J, Hubbard JL, Koepsell TD, Morris JC, Kukull WA, NIA Alzheimer's Disease Centers (2007). The National Alzheimer’s Coordinating Center (NACC) database: the Uniform Data Set. Alzheimer Dis Assoc Disord.

[14] Ogm TG, Scharmagl H, März W, Bohl J (1999). Apolipoprotein E isoforms and the development of low and high Braak stages of Alzheimer’s disease-related lesions. Acta Neuropathol.

[15] Ghebremedhin E, Schultz C, Braak E, Braak H (1998). High frequency of apolipoprotein E epsilon4 allele in young individuals with very mild Alzheimer’s disease-related neurofibrillary changes. Exp Neurol.

